# Bounds for Combinations of Toader Mean and Arithmetic Mean in Terms of Centroidal Mean

**DOI:** 10.1155/2013/842542

**Published:** 2013-10-22

**Authors:** Wei-Dong Jiang

**Affiliations:** Department of Information Engineering, Weihai Vocational College, Weihai, Shandong 264210, China

## Abstract

The authors find the greatest value *λ* and the least value *μ*, such that the double inequality $\bar{C}(\lambda a+(1-\lambda b),\lambda b+(1-\lambda )a)<\alpha A(a,b)+(1-\alpha )T(a,b)<\bar{C}(\mu a+(1-\mu )b,\mu b+(1-\mu )a)$ holds for all *α* ∈ (0,1) and *a*, *b* > 0 with *a* ≠ *b*, where $\bar{C}(a,b)=2(a^{2}+ab+b^{2})/3(a+b)$, *A*(*a*, *b*) = (*a* + *b*)/2, and $T\left(a,b\right)=\left(2/\pi \right)\int_{0}^{\pi /2}\sqrt{a^{2}{\text{cos}}^{2}\theta +b^{2}{\text{sin}}^{2}\theta }d\theta$ denote, respectively, the centroidal, arithmetic, and Toader means of the two positive numbers *a* and *b*.

## 1. Introduction

 In [1], Toader introduced a mean

(1) T(a,b)=2π∫0π/2a2cos⁡2θ+b2sin2θdθ={2aℰ1−(b/a)2π,a>b,2bℰ1−(a/b)2π,a<b,a,a=b,

where

(2) $$\begin{array}{c} ℰ=ℰ\left(r\right)=\int_{0}^{\pi /2}{\left(1-r^{2}\sin^{2}\theta \right)}^{1/2}d\theta , \end{array}$$

for *r* ∈ [0,1] is the complete elliptic integral of the second kind.

In recent years, there have been plenty of literature, such as [2–6], dedicated to the Toader mean.

For *p* ∈ ℝ and *a*, *b* > 0, the centroidal mean $\bar{C}(a,b)$ and *p*th power mean *M*_*p*_(*a*, *b*) are, respectively, defined by

(3) $$\begin{array}{c} \bar{C}\left(a,b\right)=\frac{2\left(a^{2}+ab+b^{2}\right)}{3\left(a+b\right)},\\ M_{p}\left(a,b\right)=\{\begin{array}{cc} {\left(\frac{a^{p}+a^{p}}{2}\right)}^{1/p}, & p\neq 0,\\ \sqrt{ab}, & p=0. \end{array} \end{array}$$

In [7], Vuorinen conjectured that

(4) $$\begin{array}{c} M_{3/2}\left(a,b\right)<T\left(a,b\right), \end{array}$$

for all *a*, *b* > 0 with *a* ≠ *b*. This conjecture was verified by Qiu and Shen [8] and by Barnard et al. [9], respectively.

In [10], Alzer and Qiu presented a best possible upper power mean bound for the Toader mean as follows:

(5) $$\begin{array}{c} T\left(a,b\right)<M_{\log2/\log\left(\pi /2\right)}\left(a,b\right), \end{array}$$

for all *a*, *b* > 0 with *a* ≠ *b*.

Chu et al. [5] proved that the double inequality

(6) C(αa+(1−α)b,αb+(1−α)a)  <T(a,b)  <C(βa+(1−β)b,βb+(1−β)a)

holds for all *a*, *b* > 0 with *a* ≠ *b* if and only if *α* ≤ 3/4 and $\beta \geq 1/2+\sqrt{4\pi -\pi^{2}}/(2\pi )$.

Very recently, Hua and Qi [11] proved that the double inequality

(7) αC¯(a,b)+(1−α)A(a,b)  <T(a,b)  <βC¯(a,b)+(1−β)A(a,b)

is valid for all *a*, *b* > 0 with *a* ≠ *b* if and only if *α* ≤ 3/4 and *β* ≥ (12/*π*) − 3. Where *A*(*a*, *b*) = (*a* + *b*)/2 denote the arithmetic mean.

For positive numbers *a*, *b* > 0 with *a* ≠ *b*, let

(8) $$\begin{array}{c} J\left(x\right)=\bar{C}\left(xa+\left(1-x\right)b,xb+\left(1-x\right)a\right) \end{array}$$

be on [1/2, 1]. It is not difficult to directly verify that *J*(*x*) is continuous and strictly increasing on [1/2, 1].

The main purpose of the paper is to find the greatest value *λ* and the least value *μ*, such that the double inequality $\bar{C}(\lambda a+(1-\lambda b),\lambda b+(1-\lambda )a)<\alpha A(a,b)+(1-\alpha )T(a,b)<\bar{C}(\mu a+(1-\mu )b,\mu b+(1-\mu )a)$ holds for all *α* ∈ (0,1) and *a*, *b* > 0 with *a* ≠ *b*. As applications, we also present new bounds for the complete elliptic integral of the second kind.

## 2. Preliminaries and Lemmas

 In order to establish our main result, we need several formulas and Lemmas below.

For 0 < *r* < 1 and $r^{\prime }=\sqrt{1-r^{2}}$, Legendre's complete elliptic integrals of the first and second kinds are defined in [12, 13] by

(9) $$\begin{array}{c} 𝒦=𝒦\left(r\right)=\int_{0}^{\pi /2}{\left(1-r^{2}\sin^{2}\theta \right)}^{-1/2}d\theta ,\\ 𝒦\prime ={𝒦}^{\prime }\left(r\right)=𝒦\left(r^{\prime }\right),\\ 𝒦\left(0\right)=\frac{\pi }{2},\;\;𝒦\left(1\right)=\infty ,\\ ℰ=ℰ\left(r\right)=\int_{0}^{\pi /2}{\left(1-r^{2}\sin^{2}\theta \right)}^{1/2}d\theta ,\\ ℰ\prime ={ℰ}^{\prime }\left(r\right)=ℰ\left(r^{\prime }\right),\\ ℰ\left(0\right)=\frac{\pi }{2},\;\;ℰ\left(1\right)=1, \end{array}$$

respectively.

For 0 < *r* < 1, the formulas

(10) $$\begin{array}{c} \frac{d𝒦}{dr}=\frac{ℰ-{r^{\prime }}^{2}𝒦}{r{r^{\prime }}^{2}},\;\;\frac{dℰ}{dr}=\frac{ℰ-𝒦}{r},\\ \frac{d\left(ℰ-{r^{\prime }}^{2}𝒦\right)}{dr}=r𝒦,\;\;\frac{d\left(𝒦-ℰ\right)}{dr}=\frac{rℰ}{{r^{\prime }}^{2}},\\ ℰ\left(\frac{2\sqrt{r}}{1+r}\right)=\frac{2ℰ-{r^{\prime }}^{2}𝒦}{1+r} \end{array}$$

were presented in [14, Appendix E, pages 474-475].

**Lemma 1 (see [14, Theorem 3.21(1), 3.43 exercises 13(a)])**

The function  (*ℰ* − *r*′^2^ *𝒦*)/*r* ^2^ is strictly increasing from (0,1) to (*π*/4,1), and the function 2*ℰ* − *r*′^2^ *𝒦* is increasing from (0,1) to (*π*/2,2).

**Lemma 2**

Let *u*, *α* ∈ (0,1) and

(11) fu,α(r)=13ur2 −(1−α)(2π(2ℰ(r)−(1−r2)𝒦(r))−1).

Then, *f*_*u*,*α*_ > 0, for all *r* ∈ (0,1) if and only if *u* ≥ 3(1 − *α*)(4/*π* − 1), and *f*_*u*,*α*_ < 0, for all *r* ∈ (0,1) if and only if *u* ≤ 3(1 − *α*)/4.

**Proof.**

From (11), one has

(12) $$\begin{array}{c} f_{u,\alpha }\left(0^{+}\right)=0, \end{array}$$

(13) $$\begin{array}{c} f_{u,\alpha }\left(1^{-}\right)=\frac{1}{3}u-\left(1-\alpha \right)\left(\frac{4}{\pi }-1\right), \end{array}$$

(14) $$\begin{array}{c} f_{u,\alpha }^{\prime }\left(r\right)=\frac{2}{3}r\left[u-3\left(1-\alpha \right)g\left(r\right)\right], \end{array}$$

where *g*(*r*) = (1/*π*)((*ℰ* − *r*′^2^ *𝒦*)/*r* ^2^).

We divide the proof into four cases.

*Case 1 (u* ≥ 3(1 − *α*)/*π).*  From (14) and Lemma 1 together with the monotonicity of *g*(*r*), we clearly see that *f*_*u*,*α*_(*r*) is strictly increasing on (0,1). Therefore, *f*_*u*,*α*_(*r*) > 0, for all *r* ∈ (0,1).

*Case 2 (u* ≤ 3(1 − *α*)/4*).* From (14) and Lemma 1 together with the monotonicity of *g*(*r*), we obtain that *f*_*u*,*α*_(*r*) is strictly decreasing on (0,1). Therefore, *f*_*u*,*α*_(*r*) < 0, for all *r* ∈ (0,1).

*Case 3 (*3(1 − *α*)/4 < *u* ≤ 3(1 − *α*)(4/*π* − 1)*).* From (13) and (14) together with the monotonicity of *g*(*r*), we see that there exists *λ* ∈ (0,1), such that *f*_*u*,*α*_(*r*) is strictly increasing in (0, *λ*] and strictly decreasing in [*λ*, 1) and

(15) $$\begin{array}{c} f_{u,\alpha }\left(1^{-}\right)\leq 0. \end{array}$$

Therefore, making use of (12) and inequality (15) together with the piecewise monotonicity of *f*_*u*,*α*_(*r*) leads to the conclusion that there exists 0 < *λ* < *η* < 1, such that *f*_*u*,*α*_(*r*) > 0 for *r* ∈ (0, *η*) and *f*_*u*,*α*_(*r*) < 0 for *r* ∈ (*η*, 1).

*Case 4 (*3(1 − *α*)(4/*π* − 1) ≤ *u* < 3(1 − *α*)/*π).* Equation (13) leads to

(16) $$\begin{array}{c} f_{u,\alpha }\left(1^{-}\right)\geq 0. \end{array}$$

From (13) and (14) together with the monotonicity of *g*(*r*), we clearly see that there exists *λ* ∈ (0,1), such that *f*_*u*,*α*_(*r*) is strictly increasing in (0, *λ*] and strictly decreasing in [*λ*, 1). Therefore, *f*_*u*,*α*_(*r*) > 0 for *r* ∈ (0,1) follows from (12) and (16) together with the piecewise monotonicity of *f*_*u*,*α*_(*r*).

## 3. Main Results

Now, we are in a position to state and prove our main results.

**Theorem 3.**

If *α* ∈ (0,1) and *λ*, *μ* ∈ (1/2,1), then the double inequality

(17) C¯(λa+(1−λ)b,λb+(1−λ)a)  <αA(a,b)+(1−α)T(a,b)  <C¯(μa+(1−μ)b,μb+(1−μ)a)

holds for all *a*, *b* > 0 with *a* ≠ *b* if and only if

(18) $$\begin{array}{c} \lambda \leq \frac{1}{2}+\frac{\sqrt{3\left(1-\alpha \right)}}{4},\\ \mu \geq \frac{1}{2}\left(1+\sqrt{3\left(1-\alpha \right)\left(\frac{4}{\pi }-1\right)}\right). \end{array}$$

**Proof.**

Since *A*(*a*, *b*), *T*(*a*, *b*), and $\bar{C}(a,b)$ are symmetric and homogeneous of degree one, without loss of generality, we assume that *a* > *b*. Let *p* ∈ (1/2,1), *t* = *b*/*a* ∈ (0,1), and *r* = (1 − *t*)/(1 + *t*). Then,

(19) C¯(pa+(1−p)b,pb+(1−p)a)  −αA(a,b)−(1−α)T(a,b) =a23((p+(1−p)ba)2    +(p+(1−p)ba)(pba+1−p)    +(pba+1−p)2)(1+ba)−1  −αa1+(b/a)2  −(1−α)2aπℰ(1−(ba)2) =a{23((p+(1−p)t)2     +(p+(1−p)t)(pt+1−p)     +(pt+1−p)2)(1+t)−1    −α1+t2−(1−α)2πℰ(1−t2)} =a{(1−2p)2r2+33(1+r)−α11+r   −(1−α)2π2ℰ−r′2𝒦1+r} =a1+r[13(1−2p)2r2+1−α     −(1−α)2π(2ℰ−r′2𝒦)].

Therefore, Theorem 3 follows easily from Lemma 2 and (19).

Let *α* = 1/4,  *λ* = 7/8,  $\mu =\left(1/2\right)\left(1+\left(3\sqrt{4/\pi -1}/2\right)\right)$. Then, from Theorem 3, we get new bounds for the complete elliptic integral *ℰ*(*r*) of the second kind in terms of elementary functions as follows.

**Corollary 4.**

For *r* ∈ (0,1) and $r^{\prime }=\sqrt{1-r^{2}}$, one has

(20) $$\begin{array}{c} \frac{\pi }{2}\left[\frac{5+6r^{\prime }+5{r^{\prime }}^{2}}{8\left(1+r^{\prime }\right)}\right]<ℰ\left(r\right)<\pi \left[\frac{r^{\prime }+\left(2/\pi \right){\left(1-r^{\prime }\right)}^{2}}{1+r^{\prime }}\right]. \end{array}$$

## 4. Remarks

**Remark 5.**

In the recent past, the complete elliptic integrals have attracted the attention of numerous mathematicians. In [4], it was established that

(21) π2[121+r′22+1+r′4]  <ℰ(r)  <π2[4−π(2−1)π1+r′22+(2π−4)(1+r′)2(2−1)π],

for all *r* ∈ (0,1).

Guo and Qi [15] proved that

(22) $$\begin{array}{c} \frac{\pi }{2}-\frac{1}{2}\log\frac{{\left(1+r\right)}^{1-r}}{{\left(1-r\right)}^{1+r}}<ℰ\left(r\right)<\frac{\pi -1}{2}+\frac{1-r^{2}}{4r}\log\frac{1+r}{1-r}, \end{array}$$

for all *r* ∈ (0,1).

Yin and Qi [16] presented that

(23) $$\begin{array}{c} \frac{\pi }{2}\frac{\sqrt{6+2\sqrt{1-r^{2}}-3r^{2}}}{2\sqrt{2}}\leq ℰ\left(r\right)\leq \frac{\pi }{2}\frac{\sqrt{10-2\sqrt{1-r^{2}}-5r^{2}}}{2\sqrt{2}}, \end{array}$$

for all *r* ∈ (0,1).

It was pointed out in [4] that the bounds in (21) for *ℰ*(*r*) are better than the bounds in (22) for some *r* ∈ (0,1).

**Remark 6.**

The lower bound in (20) for *ℰ*(*r*) is better than the lower bound in (21). Indeed,

(24) 5+6x+5x28(1+x)−[121+x22+1+x4] =3x2+2x+3−22(1+x2)(1+x)8(1+x),(3x2+2x+3)2−(22(1+x2)(1+x))2 =(1−x)4>0,

for all *x* ∈ (0,1).

**Remark 7.**

The following equivalence relations for *x* ∈ (0,1) show that the lower bound in (20) for *ℰ*(*r*) is better than the lower bound in (23):

(25) 5+6x+5x28(1+x)>6+2x−3(1−x2)22⇔(5x2+6x+5)2>8(x+1)2(3x2+2x+3)⇔(x−1)4>0.
